# A Plausible Proposition of CCL20-Related Mechanism in *Fusobacterium nucleatum*-Associated Oral Carcinogenesis

**DOI:** 10.3390/life11111218

**Published:** 2021-11-10

**Authors:** Devi Prasad Mandal, Neeta Mohanty, Paresh Kumar Behera, Divya Gopinath, Sasmita Panda, Abdulaziz A. Al-Kheraif, Darshan Devang Divakar, Sukumaran Anil, Swagatika Panda

**Affiliations:** 1 Institute of Dental Sciences, Siksha ’O’ Anusandhan Deemed to Be University, Bhubaneswar 751030, Odisha, India; deviprasadmandal@soa.ac.in; 2 Department of Oral Pathology and Microbiology, Institute of Dental Sciences, Siksha ’O’ Anusandhan Deemed to Be University, Bhubaneswar 751030, Odisha, India; neetamohanty@soa.ac.in; 3 Head and Neck Oncology, Acharya Harihar Regional Cancer Centre, Cuttack 753007, Odisha, India; pkb_scb@yahoo.com; 4 Clinical Oral Health Sciences Division, School of Dentistry, International Medical University, Kuala Lumpur 57000, Malaysia; divyagopinath@imu.edu.my; 5 Department of Pathology, Acharya Harihar Regional Cancer Centre, Cuttack 753007, Odisha, India; drsasmitapanda@gmail.com; 6 Dental Biomaterials Research Chair, Dental Health Department, College of Applied Medical Sciences, King Saud University, P.O. Box 10219, Riyadh 11433, Saudi Arabia; aalkhuraif@ksu.edu.sa (A.A.A.-K.); ddivakar@ksu.edu.sa (D.D.D.); 7 Department of Dentistry, Oral Health Institute, Hamad Medical Corporation, Doha P.O. Box 3050, Qatar; asukumaran1@hamad.qa; 8 College of Dental Medicine, Qatar University, Doha P.O. Box 2713, Qatar

**Keywords:** oral squamous cell carcinoma, *Fusobacterium nucleatum*, CCL20

## Abstract

Objective: The objective of this prospective observational case–control study is to evaluate the prevalence of *Fusobacterium nucleatum* in the tissues of oral squamous cell carcinoma (OSCC). Reconnoitering the CCL20-related mechanism of carcinogenesis in *Fusobacterium nucleatum*-positive OSCC is another objective. Methodology: Tissues from 50 OSCC patients and 30 healthy oral tissues were collected. The prevalence of *Fusobacterium nucleatum* was evaluated in both tumour and healthy tissue by polymerase chain reaction. The immunohistochemistry of OSCC tissues was conducted to evaluate the difference in the expression of CCL20 between *Fusobacterium nucleatum*-positive and -negative OSCC tissues. Results: *Fusobacterium nucleatum* was significantly (*p* < 0.001) prevalent in OSCC tissues (74%), compared to healthy tissues (26%). No association of *Fusobacterium nucleatum* or CCL20 immuno-expression with any clinical or histopathological features of OSCC was observed. While the intensity of CCL20 immuno-expression did not differ (*p* = 0.053), the CCL20-positive cell population was significantly different (*p* = 0.034) between *Fusobacterium nucleatum*-positive and -negative OSCC. Conclusion: *Fusobacterium nucleatum* is possibly prevalent in oral cancer tissues in the Indian population. By using immunohistochemistry, this is the first study to propose that the carcinogenesis in *Fusobacterium nucleatum*-positive OSCC may be CCL20-related. The findings enrich the knowledge of mechanisms involved in *Fusobacterium nucleatum*-mediated oral carcinogenesis.

## 1. Introduction

Malignant neoplasms that develop on the lips, mouth, nose and other head and neck sites are termed as head and neck cancers; the majority of this group is represented by oral squamous cell carcinoma (OSCC) [1]. Due to the aggressiveness and ability to metastasize, OSCC is difficult to treat with traditional therapies such as surgery and radiotherapy alone [2]. Carcinogenesis is attributed to several epigenetic, genetic and non-genetic factors. Inflammations, one of the non-genetic factors, activate transcription factors that, in turn, enhance the expression of genes common to both immuno-inflammatory responses and the survival and proliferation of cancer cells [3]. Since the characterisation of *Helicobacter pylori* as a class I carcinogen [4], carcinogenicity has been linked to bacteria. Subsequently, multiple studies have evaluated the role of bacteria in the cancer of several organs [5,6,7,8]. Owing to the presence of complex multispecies bacterial communities in the oral cavity, the hypothesis of bacteria causing oral carcinogenesis was postulated by Nagy et al. [9] and was later established by multiple studies [10,11,12,13]. The understanding of the interactions of the oral bacteria with OSCC has further evolved due to modern sequencing technologies. Although singling out carcinogenic microorganism is a formidable task, *Fusobacterium nucleatum* remains the most significant one not only in colorectal [14,15] but also in oral carcinogenesis [16,17,18]. Brennan et al. [19] have critically appraised *Fusobacterium nucleatum* as a symbiont and opportunistic oncobacterium. The suggested mechanisms of *Fusobacterium nucleatum*-mediated initiation and progression of carcinogenesis include generating a pro-inflammatory tumour microenvironment, immune evasion and immune suppression by the interaction of cell-surface molecules with host immune cells and stromal cells [20,21,22,23]. The latter may be executed through the production of cytokines and chemokines [24], including human beta-defensin (hBD) and Macrophage inflammatory protein (MIP 3 alpha/CCL20) [25]. CCL20 is a small protein that is proven to be associated with colorectal and hepatocellular carcinoma [26,27,28]. Abiko et al. in 2003 [29], suggested that CCL20 was shown to initiate and promote carcinogenesis in OSCC by controlling the immune response to bacteria such as *Actinobacillus actinomycetemcomitans* [29], as well as increasing the expression of CD163 on macrophages [30]. Very recently, *Fusobacterium nucleatum* was found to be positively associated with salivary CCL20 levels [31]. Such evidence led to hypothesizing that *Fusobacterium nucleatum*-mediated oral carcinogenesis may be CCL20-related. Therefore, the primary objective of this study is to evaluate the prevalence of *Fusobacterium nucleatum* in OSCC in the Indian population, followed by associating that with clinical and histopathological features. To evaluate the immunohistochemical expression of CCL20 in *Fusobacterium nucleatum*-positive OSCC tissues is another objective of this study.

## 2. Methodology

The study was conducted according to the STROBE guidelines for observational case–control studies. 

### 2.1. Study Design and Settings

This is a prospective observational case–control study to evaluate the immunohistochemical expression of CCL20 in *Fusobacterium nucleatum*-positive and -negative OSCC tissues. Subjects were recruited between December 2019 and December 2020. After the researchers obtained ethical approval, the study subjects were recruited from the Department of Head and Neck Oncology, Acharya Harihara Regional Cancer Centre, Odisha, India (068-IEC-AHRCC). All methods used in this study were abided by the relevant guidelines of the declaration of Helsinki on biomedical research involving human subjects. Written informed consent was obtained from each participant. 

### 2.2. Participants and Variables

Patients clinically diagnosed with OSCC were included in this study as the study group and patients reported to the institution either for removal of impacted or over-erupted third molars or flap surgery were included as the control. Patients with a history of antibiotic consumption during last 2 months were excluded. Patients with field cancerization, recurrent oral cancers and coexisting infectious diseases were also excluded. The study group and the control group comprised of 50 and 30 patients, respectively. The detailed clinical characteristics of the subjects, such as age, sex, habits and oral hygiene index, were recorded for both groups. Oral hygiene was assessed by the simplified Oral hygiene index [32].

### 2.3. Sampling and Sample Processing 

Tissue samples were procured from the control group during removal of impacted molars or flap surgery. Tissue samples from the study group were collected during the surgical resection of OSCC. Part of the tissue from each group was stored in formalin for histopathological evaluation. The other parts were immediately rinsed in normal saline and stored in a pre-filled vial containing 2 mL of nucleic acid stabilizing solution RNA*later*™ (Invitrogen, Waltham, MA, USA). The vial was stored at 4 °C for 24 h before transferring to −80 °C to allow enough time for the solution to penetrate tissues. Retromolar tissue of 0.5 cm^3^ was excised in patients undergoing third-molar extractions and handled in the same way as the cancer tissues. Samples were thawed and transferred into a microcentrifuge tube and homogenized using Polypropylene micro pestle (Tarson, London, UK). Proteinase K (QIAamp DNA Mini kit, Qiagen) was added and samples were incubated at 56 °C in a water bath with periodical vortexing until total lysis of the tissues was observed (6 h). Samples were then incubated in 200 μL of Buffer AL (QIAamp DNA Mini kit, Qiagen) for 30 min at 70 °C before continuing extraction using QIAamp DNA mini kit (Qiagen). As a next step, 200 μL of ethanol (96–100%) was added to the sample and mixed thoroughly by vortexing. The samples were loaded on a DNA spin column (Qiagen) and centrifuged at 8000 rpm in a tabletop centrifuge. The columns were then washed with Qiagen buffers AW1 and AW2 and, finally, DNA was eluted using distilled water. The DNA sample integrity was assessed by agarose gel electrophoresis on 1% agarose.

### 2.4. Polymerase Chain Reaction (PCR) Amplification

PCR was performed on extracted DNA to determine the prevalence of *Fusobacterium nucleatum* in the cases and controls using primers such as 16S rRNA-F 5′-AGA GTT TGA TCC TGG CTC AG-3′and 16S rRNA-R 5′-GTC ATC GTG CAC ACA GAA TTG CTG-3′ to amplify a 360-base pair region of the 16S rRNA gene. The amplification reaction was performed in a Thermal Cycler in a 25 mL reaction mixture containing 4.5 mL of PCR buffer (100 mM Tris- HCl, 500 mM KCl and15 mM MgCl2), 0.25 mM of each deoxynucleoside triphosphate (dNTP), 10 mM of each primer, 5 mL of NA and 1.5 units of Taq DNA polymerase (TaKaRa Bio Inc., San Jose, CA, USA) PCR was carried out for 5 min at 94 °C and 30 cycles, with each cycle consisting of denaturation at 94 °C for 30 s, annealing at 58 °C for 30 s, extension at 72 °C for 1 min and, final extension for 10 min. The amplified products were then electrophoresed on 1.5% agarose gel in Tris-acetate buffer.

### 2.5. Immunohistochemistry

Immunohistochemistry was conducted at the department of Oral Pathology and Microbiology, Institute of Dental Sciences. Formalin-fixed paraffin-embedded tissues of both study and control groups were sectioned in sizes from 3 to 5 mm, dewaxed, de-paraffinized in xylene and rehydrated through a series of graded alcohols. The samples were boiled for 15 min in a microwave oven with Proteolytic antigen retrieval reagent (pH 9.0) to increase antigen retrieval. Endogenous peroxidases were blocked by 3% hydrogen peroxidase treatment for 30 min. The slides were incubated with a primary antibody (1:50 dilution of rabbit antibody for macrophage inflammatory protein 3α (CC-chemokine cysteine motif chemokine ligand 20, CCL20; ab9829; Abcam)) overnight at 4 °C. Envision Plus detection system (Dako corporation, California, CA, USA), a biotin-free horseradish peroxidase-labelled polymer was used for detection of the antibody. The sections were developed in 3,3-diaminobenzidine and counterstained with Mayer’s hematoxylins. The slides were then dehydrated through graded alcohols and covered with coverslips. The staining intensity and percentage of CCL20-positive tumour cells were assessed manually by three experienced pathologists [33]. The staining intensity was categorized semi-quantitatively, based on the mean of positive tumour cells: 0 (<10 positive cells), 1 (10–25 positive cells), 2 (25–50 positive cells) and 3 (>50 positive cells). The positive cell population was also determined semi-quantitatively as follows: 0 (negative), 1 (weakly positive), 2 (moderately positive) and 3 (strongly positive). The average scores of three pathologists for each section were obtained in a prescribed data sheet for statistical analysis. The data were further tested for statistical significance.

### 2.6. Statistical Analysis

All the data collected were entered into the Microsoft Excel 2007 software and further analyzed in SPSS version 24 (IBM Inc., Chicago, IL, USA). All the categorical variables were expressed in terms of number/frequency and percentages. Association between two categorical variables was obtained by using the chi-square test. All the continuous variables were expressed in terms of mean and standard deviation. The significance level in the comparison of means was obtained by the independent sample *t*-test test/Mann–Whitney U test, depending on the distribution of data. A *p*-value less than 0.05 was considered statistically significant. 

## 3. Results

### 3.1. Demographics

Detailed clinical and demographic characteristics are presented in Table 1. The mean age of the cases was higher than that of the control group (*p* < 0.05). There were no statistically significant differences among oral hygiene and tobacco consumption between cases and controls. The mean age of the study participants was 51.70 ± 13.14 years with a minimum of 28 years and a maximum of 85 years. The male-to-female ratio was found to be 2.57:1. Out of 50 patients, 48 (96%) were Hindu. The distribution according to socio-economic status (SES) showed that the majority belonged to the middle SES (80%) followed by lower and upper SES. Out of all the patients with OSCC, the majority (96%) had an addiction to either tobacco or alcohol or both. Buccal mucosa and tongue shared almost equal site predilection followed by the rest. Almost half of the patients presented the T2 stage (46%), followed by the T3 (24%), T4 (18%) and T1 (12%) stages. Similarly, the predominant stage of lymph node involvement was N2 (46%). In total, 66% of the cases were well differentiated, while the rest were moderately differentiated (32%) and poorly differentiated (2%). The detailed clinical and histopathological characteristics of the subjects are provided in Table 2.

### 3.2. Prevalence of Fusobacterium Nucleatum in OSCC and Clinicopathologic Correlation

In patients with OSCC, the prevalence of *Fusobacterium nucleatum* was 74%, whereas prevalence was only 26% in the control group. This difference was statistically significant (*p* < 0.001). We did not find any significant association of *Fusobacterium nucleatum* prevalence with any clinical features such as age, gender, site, habits, staging and histopathological grading (Table 3).

### 3.3. CCL 20 Immuno-Expression and Clinicopathologic Association

**Figure 1:**
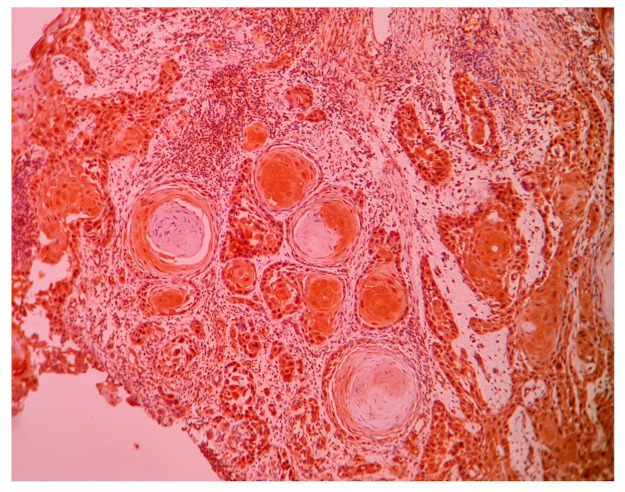
Photomicrograph (10X) showing positive immuno-expression of CCL20 in well-differentiated squamous cell carcinoma.

**Figure 2:**
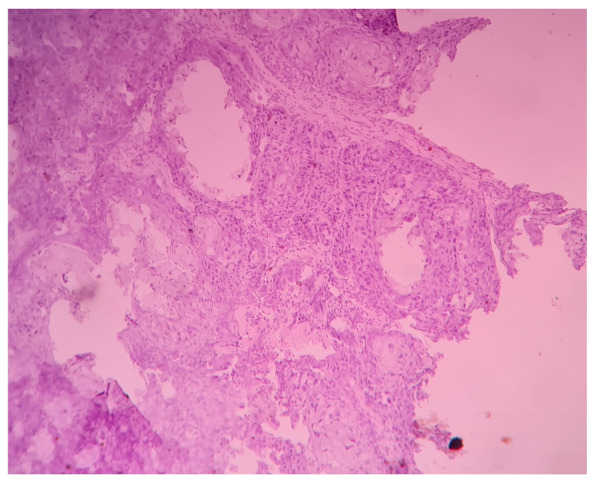
Photomicrograph (10X) showing negative immuno-expression of CCL20 in well-differentiated squamous cell carcinoma.

Only two tissues out of thirty (6.7%) in the control group were showing mild positivity, whereas 37 out of 50 cases (74%) in the study population were showing positive immuno-expression of CCL20. The intensity of CCL20 immuno-expression was found to be low in 23 tissues and moderate to high in 27 tissues (Figure 1). Negative immunoexpression of CCL20 is shown in Figure 2. A positive cell population was observed in 24 out of 50 cases. There was no significant association of CCL20 immuno-expression with any clinical or histopathological features. The power of the study as calculated by a post hoc test with the given *Fusobacterium*-positive tissues versus *Fusobacterium*-negative tissues was found to be 0.86 at an alpha error of 0.05 and effect size of 0.8 (G * power 3.1).

### 3.4. Association of CCL20 Immuno-Expression and Fusobacterium Nucleatum Prevalence

While the intensity of CCL20 immuno-expression did not show any significant difference (*p* = 0.053) between *Fusobacterium nucleatum*-positive and -negative OSCC, the CCL20-positive cell population was significantly different (*p* = 0.034) in both groups (Table 4).

## 4. Discussion

*Fusobacterium nucleatum* is a filamentous, non-spore-forming and nonmotile Gram-negative anaerobic bacterium that is commonly found in the microflora of the oral cavity and gastrointestinal tract [34]. It is often associated with periodontal [35] and inflammatory bowel diseases [36,37]. The abundance of this bacterium has been consistently found in OSCC patients in comparison to healthy controls as found via metagenomics sequencing of tumour tissues as well as saliva [16,38]. This study, conducted on the Odisha population of India, revealed a prevalence of 74% in OSCC tissues, which is much higher than the findings of the meta-analysis [39], which showed a prevalence of 16% in tumour tissues and 10% in non-tumour lesions. This augmented prevalence of *Fusobacterium nucleatum* in our study may be an attribute of tobacco-exposed tissues. *Fusobacterium nucleatum*, by the virtue of altering the redox potential and oxygen tension of the ecosystem [40] creates a favorable environment for the colonization of other anaerobic microorganisms such as *Porphyromonas gingivalis* [41], *Candida albicans* [42], etc. These anaerobes may then further damage the mucosa through the production of metabolites such as ammonia, short-chain fatty acids and sulphur compounds, which can boost the oncogenic effects of tobacco.

No significant association of *Fusobacterium nucleatum* with clinicopathological features of OSCC was established in this study. There are no studies to substantiate the clinicopathologic association of *Fusobacterium nucleatum*, though *Fusobacterium nucleatum periodontium* was found to be progressively increased from stage I to stage IV in oral cancer [42,43,44,45]. 

The smaller sample size (*n* = 50) in this study compared to the 197 samples in the previous study [29,43,44,45].

This may have caused the absence of this association. Understanding the *Fusobacterium nucleatum*-initiated mechanism of initiation and progression of OSCC is required for the development of novel approaches to treat or prevent *Fusobacterium nucleatum*-positive OSCC. A complex interplay among *Fusobacterium nucleatum* and the various immune mediators in the tumour microenvironment is currently of great interest relatively to several cancers [43,44,45,46] among which the inflammation-mediated up-regulation of cytokines and chemokines stands prominent [29,43,44,47]. These findings prompted us to investigate the immuno-expression status of CCL20 in *Fusobacterium nucleatum*-associated OSCC tissues to postulate the possibility of CCL20-related mechanisms. Before this, we compared the immuno-expression of CCL20 between the control and study group. There was a significant difference between the CCL20 immunopositive cell populations of these two groups. This finding is well supported by Chang et al. [48]. The difference in intensities of the immunoexpression of CCL20 approaches significance, which may be partly attributed to the sample size and the inherent subjectivity involved in manually interpreting the intensity of immunoexpression. However, there was no significant correlation between CCL20 immuno-expression and staging and histopathological grading. Although the immunohistochemical expression of CCL20 was also characterized as a prognostic marker in several cancers [49,50], the lack of studies on OSCC revealed controversial findings. While Ueda et al. concluded a positive correlation between salivary CCL20 and a pathological stage [31], Chang et al. found no correlation with any clinical and histopathological features except pathological lymph node metastasis [48]. The difference in nature and size of the sample as well as the techniques adopted in this handful of studies, led to the inconsistent findings. Therefore, the possibility of considering CCL20 as a prognostic marker should be well explored. This study reveals a significant difference in the prevalence of CCL20-positive cell population in *Fusobacterium nucleatum*-positive tissues, compared to *Fusobacterium nucleatum*-negative tissues (*p* = 0.034). The expression of both in vivo and in vitro CCL20 mRNA has been already evidenced by Abiko et al. in 2003, who suggested that CCL20 contributes to the oral immune response to bacterial infections such as *Actinobacillus actinomycetemcomitans* and may be involved in the progression of OSCC [29]. CCL20 exhibits chemoattractant properties towards leukocytes and, along with its receptor CCR6, it facilitates the recruitment of immature dendritic cells to the inflammatory microenvironment. *Fusobacterium nucleatum* and its cell wall extract (*Fn*CW) have been shown to stimulate human oral epithelial cells to secrete CCL20, where CCL20-inducing factors were identified as the iso-electric focusing (IEF) fraction, a component of *Fn*CW [25]. *Fusobacterium nucleatum*-associated beta-defensin inducer peptide (FAD-I), another component of *Fn*CW, was also shown to contribute towards CCL20 induction, though in negligible quantity [25].

These study findings are limited by a few factors. First, the small sample size is due to the restricted duration of the study. Second, the cross sectional nature of this study may have caused a transient association of microbiological dysbiosis in OSCC. Because of the well-established association of that with periodontitis [51] and tobacco usage [52,53], the *Fusobacterium nucleatum* prevalence in the cases may be attributed to the majority of participants having bad oral hygiene and tobacco habits. Addressing the second objective, we may suggest that the initiation and progression of *Fusobacterium nucleatum*-associated OSCC may be CCL20-related. *Candida albicans* and *Porphyromonas gingivalis*, which also contribute to oral carcinogenesis through similar mechanisms [54,55], were not studied here. This may be another confounder bias in this study. Therefore, the findings of the present study may be considered as a preliminary result and this hypothesis can be extrapolated in a larger sample. Other possible mechanisms of *Fusobacteria*-mediated initiation and progression of OSCC, such as interference in the cell adhesion process, cell cycle and epithelial–mesenchymal transition through the pathogenic membrane-associated proteins FadA/Fap2/RadD [11,43,56], need to be extrapolated further. 

## 5. Conclusions

This study evaluates the prevalence of *Fusobacterium nucleatum* in oral cancer tissues in the Odisha state, India. By using immunohistochemistry, this is the first study to propose that the carcinogenesis in *Fusobacterium nucleatum*-positive OSCC may be CCL20-related. The findings enrich the knowledge of the mechanisms involved in *Fusobacterium nucleatum*-mediated oral carcinogenesis, particularly in the Indian population. Further exploration into this matter in a larger sample size that includes various ethnicities would be highly relevant for obtaining conclusive evidence, which would impact the design of CCL20-targeted therapies in *Fusobacterium nucleatum*-positive OSCCs.

## Figures and Tables

**Table 1:** Clinical characteristics of the study participants.

Variables	Case (*n* = 50)	Control (*n* = 30)
Age (Mean) *	51.7	49.5
**Gender** Male Female	36 14	18 12
Habit ** No addiction Only tobacco Only alcohol Both tobacco and alcohol	2 31 1 16	1 6 0 23
**Site** Buccal mucos ATongue Gingivo-buccal sulcus The floor of the mouth Retromolar triagone Alveolus Palate	12 11 5 3 10 7 2	18 12
Oral hygiene ** Good Fair Bad	3 30 17	3 23 4
Peridontal status ** Mild periodontitis Moderate periodontitis Severe periodontitis	2 34 14	3 25 2

* *p* < 0.05, (*t*-test); ** *p* > 0.05 (chi-square test).

**Table 2:** Clinical and histopathologic characteristics of the cases (*n* = 50).

Variables	Frequencies	Percentages
**Gender** Male Female	36 14	72.0 28.0
**Religion** Hindu Muslim Christian	48 1 1	96.0 2.0 2.0
**Habit** No addiction Only tobacco Only alcohol Both tobacco and alcohol	2 31 1 16	4.0 62.0 2.0 32.0
**Site** Buccal mucos ATongue Gingivo-buccal sulcus The floor of the mouth Retromolar triagone Alveolus Palate	12 11 5 3 10 7 2	24.0 22.0 10.0 6.0 20.0 14.0 4.0
**Tumour stage** T1 T2 T3 T4	6 23 12 9	12.0 46.0 24.0 18.0
**Lymph node involvement** N0 N1 N2 N3	10 23 11 6	20.0 46.0 22.0 12.0
**Histopathology Grading** Well Moderate Poor	33 16 1	66.0 32.0 2.0

**Table 3:** Association of prevalence of *Fusobacterium nucleatum* in case and control.

*Fusobacterium nucleatum*	Cases (*n* = 50) N (%)	Control (*n* = 30) N (%)	*p*-Value
Present	37 (74.0)	9 (30.0)	<0.001
Absent	13 (26.0)	21 (70.0)	

**Table 4:** Association of CCL20 intensity and CCL20-positive cell population with the presence of *Fusobacterium nucleatum*. (Chi-square test was used.)

CCL20 Intensity	*Fusobacterium Nucleatum* Present (*n* = 37) N (%)	*Fusobacterium Nucleatum* Absent (*n* = 13) N (%)	*p*-Value
Positive Negative	17 (45.9) 20 (54.1)	10 (77.0) 3 (23.0)	0.053
CCL20 cell population			
<25% >25%	16 (43.2) 21 (56.7)	10 (77.0) 3 (23.0)	0.034
